# Simvastatin provides long‐term improvement of left ventricular function and prevents cardiac fibrosis in muscular dystrophy

**DOI:** 10.14814/phy2.14018

**Published:** 2019-03-26

**Authors:** Min J. Kim, Kenneth L. Bible, Michael Regnier, Marvin E. Adams, Stanley C. Froehner, Nicholas P. Whitehead

**Affiliations:** ^1^ Department of Physiology & Biophysics University of Washington Seattle Washington; ^2^ Department of Bioengineering University of Washington Seattle Washington

**Keywords:** Cardiac function, fibrosis, muscular dystrophy, simvastatin

## Abstract

Duchenne muscular dystrophy (DMD), caused by absence of the protein dystrophin, is a common, degenerative muscle disease affecting 1:5000 males worldwide. With recent advances in respiratory care, cardiac dysfunction now accounts for 50% of mortality in DMD. Recently, we demonstrated that simvastatin substantially improved skeletal muscle health and function in *mdx* (DMD) mice. Given the known cardiovascular benefits ascribed to statins, the aim of this study was to evaluate the efficacy of simvastatin on cardiac function in *mdx* mice. Remarkably, in 12‐month old *mdx* mice, simvastatin reversed diastolic dysfunction to normal after short‐term treatment (8 weeks), as measured by echocardiography in animals anesthetized with isoflurane and administered dobutamine to maintain a physiological heart rate. This improvement in diastolic function was accompanied by increased phospholamban phosphorylation in simvastatin‐treated mice. Echocardiography measurements during long‐term treatment, from 6 months up to 18 months of age, showed that simvastatin significantly improved in vivo cardiac function compared to untreated *mdx* mice, and prevented fibrosis in these very old animals. Cardiac dysfunction in DMD is also characterized by decreased heart rate variability (HRV), which indicates autonomic function dysregulation. Therefore, we measured cardiac ECG and demonstrated that short‐term simvastatin treatment significantly increased heart rate variability (HRV) in 14‐month‐old conscious *mdx* mice, which was reversed by atropine. This finding suggests that enhanced parasympathetic function is likely responsible for the improved HRV mediated by simvastatin. Together, these findings indicate that simvastatin markedly improves cardiac health and function in dystrophic mice, and therefore may provide a novel approach for treating cardiomyopathy in DMD.

## Introduction

Duchenne muscular dystrophy (DMD) is a severe, degenerative muscle disease caused by the absence of dystrophin, a large protein that links the cytoskeleton to the surface membrane in muscle cells. Loss of dystrophin causes widespread effects on muscle signaling and metabolic pathways, which culminate in muscle damage, chronic inflammation and the progressive replacement of functional muscle fibers with fibrotic connective tissue (Allen et al. 2016). These cellular and pathological changes manifest as profound muscle weakness and gradual loss of mobility over several years. Consequently, DMD boys are usually wheelchair dependent by their early teenage years and mortality commonly occurs by age 20 to 30. Until recently, respiratory weakness was the leading cause of mortality in DMD, however with the advent of enhanced ventilatory care, and subsequently a longer lifespan, cardiac complications are becoming increasingly more prevalent and are now responsible for approximately 50% of deaths in DMD patients (Mosqueira et al. 2013).

Cardiac disease in DMD is characterized by early diastolic dysfunction, which later progresses to dilated cardiomyopathy (Judge et al. 2011). In DMD boys as young as 10 years of age, presymptomatic signs of cardiac dysfunction can be detected by echocardiography using the myocardial performance index (MPI), a global measure of left ventricular systolic and diastolic function (Shabanian et al. 2011). Interestingly, at the same time, measures of systolic function such as percent ejection fraction (EF%) and fractional shortening (FS%) are not different between DMD and age‐matched control subjects (Shabanian et al. 2011). This demonstrates that MPI is a sensitive echocardiographic measure of early cardiac dysfunction in young DMD boys. The utility of MPI has also been demonstrated in children with idiopathic dilated cardiomyopathy, where it was shown to be an independent, predictive measure of mortality (Azevedo et al. 2008). In *mdx* mice, an animal model of DMD, MPI is significantly increased by 12 months of age compared to WT mice, indicating left ventricular dysfunction (Adamo et al. 2010). As with young DMD patients, *mdx* mice do not display systolic dysfunction at this age since FS% and EF% values are normal (Fayssoil et al. 2013).

Another process contributing to early cardiac disease in DMD is abnormal regulation of the autonomic nervous system, with increased sympathetic drive and decreased parasympathetic tone (Smith et al. 2014). These perturbations in autonomic control manifest as increased resting heart rate and decreased heart rate variability (HRV), as measured by ECG recordings in DMD patients (Thomas et al. 2015). This study also showed a correlation between low HRV and increased myocardial fibrosis (Thomas et al. 2015), a key pathological process that leads to functional impairment in both cardiac and skeletal muscle of DMD patients (Desguerre et al. 2009; Mosqueira et al. 2013). Interestingly, two of the most commonly used drugs to treat cardiac disease in DMD, *β*‐blockers and ACE inhibitors, decreased the average heart rate but not the HRV (Thomas et al. 2015). This data implies that sympathetic inhibition is likely not responsible for the low HRV in DMD and evidence indicates that attenuated parasympathetic drive is responsible (Lanza et al. 2001). These findings also highlight the complexity of cardiac disease in DMD and the need to find novel therapeutics for treating the early processes that initiate the progression of dilated cardiomyopathy. Currently, corticosteroids such as prednisone and deflazacort are the most widely used treatment for DMD. These drugs slow skeletal muscle disease progression only marginally and have major side‐effects (Malik et al. 2012)**.** While there is evidence that steroids provide some benefits for cardiac muscle function in DMD, a recent study cautions that their administration to patients in early childhood (<5 years of age) may increase the risk of earlier development of cardiomyopathy (Kim et al. 2017). Therefore, identifying a new drug that can be administered in the early stages of the disease, provides improvement of both skeletal and cardiac muscle function, and has minimal side‐effects, would be a major advancement for the long‐term treatment of DMD.

Recently, we reported that simvastatin, a commonly used HMG CoA‐reductase inhibitor (statin), provided a dramatic improvement in skeletal muscle function of *mdx* mice (Whitehead et al. 2015). Statins are one of the most commonly prescribed classes of drugs for treating cardiovascular disease worldwide and were originally designed to lower circulating LDL cholesterol levels. However, numerous recent reports indicate that a wide‐range of additional benefits may be ascribed to cholesterol‐independent or ‘pleiotropic’ benefits of statins, such as countering oxidative stress, inflammation, and fibrosis (Antonopoulos et al. 2012; Tanaka et al. 2013). Interestingly, all of these deleterious pathways are known to be major contributors to the pathogenesis of DMD (Allen et al. 2016) and we showed that simvastatin significantly reduced each of these processes in skeletal muscles of *mdx* mice (Whitehead et al. 2015). To date, statins have not been considered as a treatment for DMD or, indeed, any other neuromuscular disease. This may, in part, be due to the perceived risk of muscle‐related side‐effects often discussed with statin use in the general population. However, our recent paper clearly shows that simvastatin provides significant improvements in the pathophysiology of *mdx* skeletal muscle (Whitehead et al. 2015), indicating that this concern is unlikely to be pertinent to severe muscle diseases such as DMD. Given the plethora of reported benefits to cardiovascular health provided by statins, we aimed to determine whether simvastatin could offer long‐term improvement of cardiac function in *mdx* mice. Importantly, we showed by echocardiography that simvastatin significantly enhanced diastolic function both in the short and long‐term, and prevented the deposition of myocardial fibrosis. We also revealed by ECG measurements that simvastatin improved autonomic regulation of the heart, by enhancing parasympathetic activity, which led to an increase in HRV. Together, our findings reveal for the first time that simvastatin mediates a significant improvement in left ventricular physiological function and prevents the progression of cardiac muscle fibrosis in muscular dystrophy. These findings provide further impetus for clinical evaluation of simvastatin as a therapeutic with the rare potential to improve both skeletal and cardiac muscle function in DMD.

## Methods

### Ethical approval

All experimental procedures performed on mice were approved by the Institutional Animal care and Use Committee of the University of Washington (approval reference number 3298‐02). Male dystrophin‐deficient (*mdx*) and wild type (WT) mice on the C57BL/10ScSn background were used for all experiments.

### Simvastatin treatment

Simvastatin powder (TCI America) was formulated into a standard rodent diet (D12450B; Research Diets) at a concentration of 40 or 80 mg/kg as previously described (Whitehead et al. 2015). Preliminary studies indicate that these doses equate to an average of 3.5 and 6.5 mg/kg per day of simvastatin per mouse for the 40 and 80 mg/kg doses, respectively (Whitehead et al., unpublished). This equates to a dose range of ~20 to 40 mg per day for a 10‐year‐old DMD boy weighing 30 kg (Shabanian et al. 2011), based on mouse to human equivalence calculations (Reagan‐Shaw et al. 2008). This is within the recommended dose range of statins for children (O'Gorman et al. 2009). WT mice were given the same standard diet without Simvastatin. We did not use WT mice treated with simvastatin in these studies since our previous paper showed no significant effect of simvastatin on skeletal muscle function of WT mice (Whitehead et al. 2015).

### Echocardiography

Echocardiography was performed using the Vevo Strain 2100 system equipped with a high‐frequency (18–38 MHz) MS400 probe (Visual Sonics). Mice were anesthetized with isoflurane mixed with O_2_. Induction of anesthesia required 5% isoflurane for WT and 3% for *mdx*, based on preliminary observations that *mdx* is more sensitive to higher induction concentrations. Mice were then placed on the heated stage, and anesthesia was maintained throughout echocardiography by 1.5% isoflurane (1 L/min), delivered via a nose cone, for all mice. Core body temperature was maintained at ~37°C, and monitored via a rectal probe. Hair on the chest was removed (Nair) and conductive gel placed on the skin. The four limbs were taped onto the conductive surfaces on the stage. Since isoflurane depresses heart rate, Dobutamine was injected I.P (2 mg/kg) to reestablish a more normal physiological heart rate of between 600 and 650 bpm (see Table 1). Myocardial Performance Index (MPI), a global measure of left ventricular systolic and diastolic function was measured in pulsed wave Doppler mode. Here, the probe was positioned near the junction of the mitral and aortic valves, using the apical four chamber orientation. MPI was calculated by the equation: MPI = (IVRT + IVCT)/AET), where IVRT = Isovolumetric relaxation time, IVCT = Isovolumetric contraction time, and AET = Aortic ejection time. The short‐axis view was used to measure diastolic function in Tissue Doppler Mode, via the E’/A’ ratio, which measures the early diastolic filling velocity (E’) divided by the atrial peak velocity (A’). Here, the probe was positioned over the left ventricular posterior wall. Normal functioning hearts have an E’/A’ ratio > 1.0, while this value drops below 1.0 during the early stages of diastolic dysfunction. The short axis view in M‐Mode was also used to measure the percent Fractional Shortening (FS%) and Ejection Fraction (EF%) during the cardiac cycle.

**Table 1:** Echocardiography measurements of systolic function in 14‐month‐old mice

	*mdx* Con	*mdx* Sim	WT	*mdx* Con v *mdx* Sim *P* Value	*mdx* Con v WT *P* Value	*mdx* Sim v WT *P* Value
*n*	6	7	6			
Body weight	34.2 ± 0.8	33.8 ± 0.6	36.5 ± 0.7	NS	*P < 0.05*	*P < 0.05*
Heart rate	627 ± 8	651 ± 5	638 ± 19	NS	NS	NS
FS (%)	45.2 ± 0.9	45.3 ± 1.0	47.0 ± 1.5	NS	NS	NS
EF (%)	78.1 ± 1.0	78.0 ± 0.9	79.4 ± 1.6	NS	NS	NS

Echocardiography parameters and statistical differences for *mdx* Con (*n* = 6), *mdx* Sim (*n* = 7) and WT (*n* = 6) as measured at the end of the experiment (after 8 weeks of Simvastatin treatment). Note that % fractional shortening (FS%) and ejection fraction (EF%) were not statistically different between any group. Values are mean (±SEM).

### Western blotting

Western blotting was performed according to standard procedures as previously described (Whitehead et al. 2015). Briefly, muscles were homogenized in a PBS buffer containing EDTA (5 mmol/L), protease (Thermo Scientific) and phosphatase (Roche) inhibitor cocktails, and 1% Triton X‐100. Samples were loaded onto 4–20% gradient gels (Bio‐Rad) and transferred to PVDF membranes (Millipore). Membranes were blocked for 1 to 2 h with 5% skim milk in PBST or 2.5% BSA in PBST for phosphorylated proteins, and then incubated with primary antibodies in blocking buffer for 1 h at room temperature or overnight at 4°C. Primary antibodies used were; p‐PLB‐S16 (Millipore Cat# 07‐052, RRID:AB_310352), PLB (Cell Signaling Technology Cat# 8495S, RRID:AB_10949105), SERCA2 ATPase (Sigma‐Aldrich Cat# S1314, RRID:AB_261436) and GAPDH (Santa Cruz Biotechnology Cat# sc‐25778, RRID:AB_10167668). HRP‐labeled secondary antibodies (Jackson ImmunoResearch) were incubated for 1 h at room temperature. Bands were detected using a FluorChem M imaging system (Protein Simple).

### ECG studies

ECG was measured in vivo in conscious mice using a surgically implanted telemetry device (ETA‐F10, DSI) in 14‐month‐ old WT mice and *mdx* mice with or without 8 weeks of Simvastatin treatment (80 mg/kg). Mice were anesthetized using isoflurane and the telemetry device was implanted subcutaneously in the abdomen with leads placed diagonally to the heart according to the manufacture's recommendations. Mice were given the analgesic buprenorphine (0.05 mg/kg I.P.) before and after surgery.

ECG measurements were performed after at least 3 days of recovery from surgery. A baseline ECG measurement was measured over 45 to 60 s when mice were resting in the cage to avoid movement artifacts in the ECG recordings. If the mice began moving during the recording, another measurement was taken after they had resumed a resting posture. In order to evaluate the role of the parasympathetic nervous system on ECG measurements, mice were injected I.P with the parasympathetic blocker atropine (2 mg/kg). ECG data was recorded on the telemetry software and raw values were exported as text files into a spreadsheet (Excel). Analysis of the raw ECG recordings was performed using a software package (Kubios HRV). For each recording, all R waves were labeled and the software measured the time (ms) between all R‐R intervals, which was used to calculate HRV as the average SD of R‐R intervals as well as average heart rate (bpm).

### Fibrosis quantification

Previously, we showed that fibronectin immunostaining provided a reliable, quantitative measure of *mdx* diaphragm fibrosis (Whitehead et al. 2015). Therefore, we used this method in the current study to quantify cardiac fibrosis. Muscles were embedded in O.C.T compound and frozen in isopentane cooled by liquid nitrogen. Cryosections (10 *μ*m thick) were placed on a glass side. Sections were fixed with 2% paraformaldehyde for 10 min, and permeabilized with 0.1% Triton X‐100 for 5 min. Sections were then incubated in blocking buffer (0.8% BSA and 1% fish gelatin in PBS) for 45 min. The rabbit fibronectin primary antibody (Sigma‐Aldrich Cat# F3648, RRID:AB_476976) was added for 1 hr 30 min at room temperature. The secondary antibody (AlexaFluor488 conjugated), and DAPI (nuclear stain) were then added for 1 h 30 min. Sections were mounted with anti‐fade Gold reagent (Life Technologies) and imaged with a Zeiss Axioscop 2 fluorescent microscope. Images were converted to grayscale in Image J and a threshold applied to quantify the area of fluorescence as a percent of the total area of the section, as previously described (Whitehead et al. 2015).

### Statistical analysis

All data are presented as the mean ± S.E.M. The significance level for all experiments was set at *P *<* *0.05. Comparisons between groups were analyzed using a one‐way ANOVA or two‐way ANOVA for time‐dependent data. The analysis program used was Data Desk (Ithaca, NY, USA).

## Results

### Simvastatin reverses diastolic dysfunction in *mdx* mice and increases Phospholamban phosphorylation

**Figure 1:**
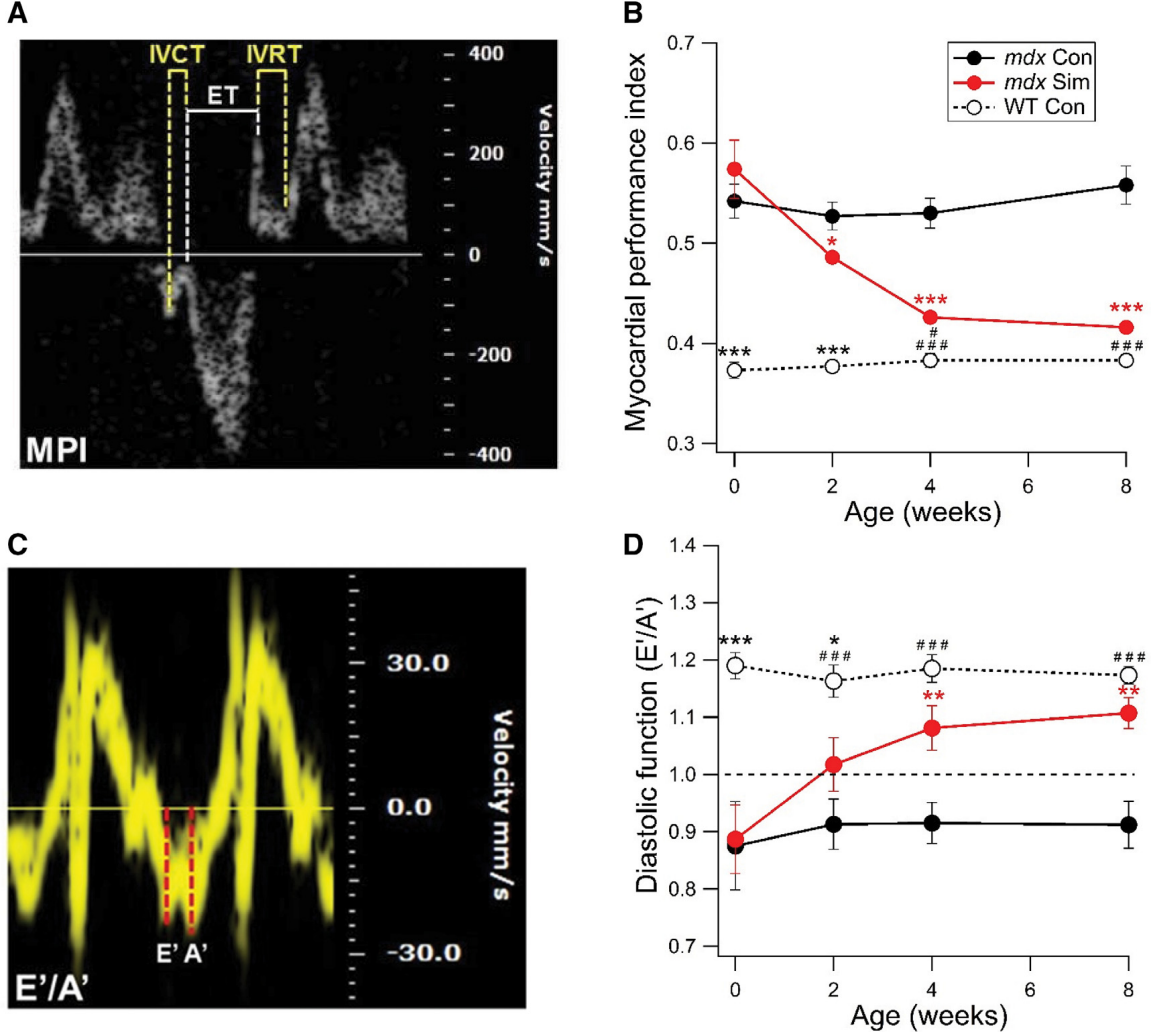
Short‐term simvastatin treatment of old *mdx* mice reverses left ventricular diastolic dysfunction. In these experiments, *mdx* mice were treated with Simvastatin (80 mg/kg food) starting at 12 months of age for a total of 8 weeks. (A) Representative recording showing the method of analysis for the myocardial performance index (MPI). (B) Pooled data showing the changes in MPI over time. The first measurement was taken when mice were 12 months of age (Time, 0 weeks). *P <* *0.05 versus *mdx* Con, ***P <* *0.001 versus *mdx* Con, ***P <* *0.001 versus *mdx* Con and *mdx* Sim, ^#^ *P *<* *0.05 versus *mdx* Sim, ^###^ *P *<* *0.001 versus *mdx* Con. (C) Representative recording showing the method of analysis for the E’/A’ ratio, a measure of diastolic function (D) Pooled data showing the changes in the E’/A’ ratio over time. Note that a ratio > 1.0 is considered a normal value (dotted line). **P <* *0.01 versus *mdx* Con, *P <* *0.05 versus *mdx* Sim, ***P <* *0.001 versus *mdx* Con and *mdx* Sim, ^###^ *P *<* *0.001 versus *mdx* Con. WT (*n* = 6), *mdx* Con (*n* = 6), *mdx* Sim (*n* = 7).

In the first series of experiments, we tested if short‐term (8 weeks) simvastatin treatment could improve cardiac function in old *mdx* mice, starting at 12 months of age, at which time skeletal muscle degeneration is more advanced (Whitehead et al. 2015) and diastolic cardiac dysfunction is evident by echocardiography, as evaluated by MPI (Fig. 1A) (Adamo et al. 2010). Remarkably, after only 2 weeks of simvastatin treatment (80 mg/kg food), comparable to a daily human dose of 20–40 mg (Whitehead et al. 2015), *mdx* Sim mice already showed a significant reduction in MPI compared to *mdx* Con mice (Fig. 1B). MPI values decreased further toward WT after 4 weeks of simvastatin treatment and by 8 weeks, *mdx* Sim values were not significantly different to WT. Over the same period, MPI values for *mdx* Con mice remained unchanged (see Fig. 1B). We also assessed diastolic function by using tissue Doppler imaging to determine the ratio of the early diastolic velocity (E’) to peak atrial velocity (A’), as shown in Figure 1C. The E’/A’ ratio was previously shown to be <1.0 in 12‐month‐old *mdx* mice, indicating diastolic dysfunction (Adamo et al. 2010). Here, the E’/A’ ratio was <1.0 in *mdx* Con mice but this value increased above 1.0 in *mdx* Sim mice and was significantly higher than *mdx* Con after 4 and 8 weeks of simvastatin treatment (Fig. 1D). Values for *mdx* Sim were significantly different to WT at 0 and 2 weeks, but were not significantly different at 4 and 8 weeks after simvastatin treatment (see Fig. 1D). Interestingly, commonly used echocardiographic measures of systolic function, FS% and EF%, were not different between any of the groups (Table 1), consistent with the findings in young DMD boys (Shabanian et al. 2011). Together, this data indicates that Simvastatin reversed left ventricular dysfunction in *mdx* mice, primarily by improving the abnormal diastolic function (Judge et al. 2011).

**Figure 2:**
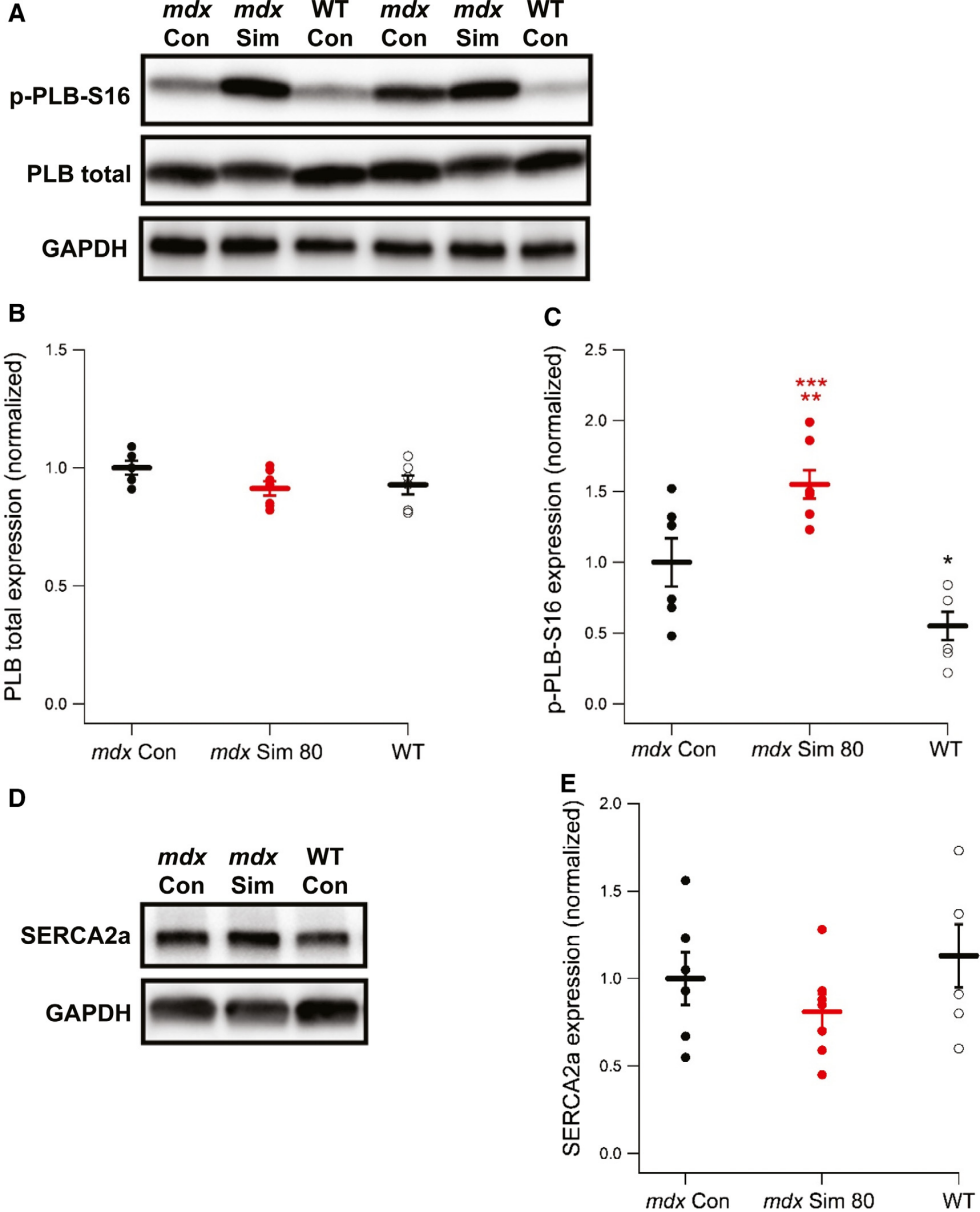
Simvastatin increases expression of p‐PLB‐S16, a key regulator of SERCA2a activity. (A) Representative Western blot showing the expression of Phospholamban phosphorylated at serine 16 (p‐PLB‐S16) and total Phospholamban (PLB total) from cardiac muscle of 14‐month‐old WT mice and *mdx* mice with or without 8 weeks of simvastatin treatment. Pooled data showing individual values and the mean expression levels of (B) PLB total and (C) p‐PLB‐S16. **P <* *0.01 versus *mdx* Con, ***P <* *0.001 versus WT, *P <* *0.05 versus *mdx* Con. Data are shown as the mean ± SEM. WT (*n* = 6), *mdx* Con (*n* = 6), *mdx* Sim (*n* = 7). (D) Representative Western blot showing the expression of SERCA2a. (E) Pooled data showing individual values and the mean expression levels of SERCA2a.

Phospholamban (PLB) is a major inhibitor of the sarcoplasmic/endoplasmic reticulum Ca^2+^ ATPase 2a (SERCA2a) activity in the heart and can therefore regulate intracellular resting calcium and diastolic function. Unphosphorylated PLB binds SERCA and inhibits its activity, while phosphorylation at serine 16 (p‐PLB‐S16) relieves the inhibition of SERCA and enhances activity of the Ca^2+^ pump (MacLennan and Kranias 2003). Here we used immunoblotting to demonstrate that p‐PLB‐S16 was significantly increased by 55% following Simvastatin treatment compared to *mdx* Con, while total PLB (PLB) was not different between the groups (Fig. 2A–C). We also measured the levels of SERCA2a expression, which were not significantly different between the three groups of mice (Fig. 2D,E).

### Simvastatin treatment increases heart rate variability by enhanced parasympathetic function in *mdx* mice

**Figure 3:**
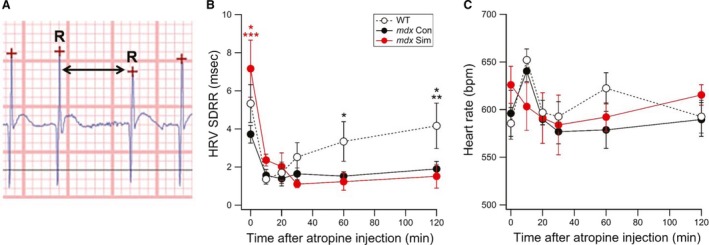
Short‐term simvastatin treatment of old *mdx* mice increases HRV by enhancing parasympathetic activity. ECG was assessed using an implanted telemetry device in WT,* mdx* Con and *mdx* Sim mice following 8 weeks of simvastatin (80 mg/kg food) treatment. (A) Representative ECG trace showing the R waves (crosses), which were then used to calculate R‐R time intervals (arrow). Measurements were recorded at baseline (Time 0) and up to 120 min after I.P. injection of atropine. (B) Pooled data showing the HRV, as measured from the standard deviation of R‐R intervals (SDRR). *P <* *0.05 versus WT, ***P <* *0.001 versus *mdx* Con, *P <* *0.05 versus *mdx* Con and *mdx* Sim, **P <* *0.01 versus *mdx* Sim, *P <* *0.05 versus *mdx* Con. (C) Pooled values showing the average heart rate. WT (*n* = 6), *mdx* Con (*n* = 7), *mdx* Sim 80 (*n* = 7).

DMD patients show evidence of autonomic dysfunction, which is detected by ECG as an increased heart rate and decreased heart rate variability (HRV) (Thomas et al. 2015). In the present study, short‐term ECG was recorded in old (14 months of age) WT, *mdx* Con and *mdx* Sim 80 mice, using a surgically implanted ECG telemetry device. Measurements at each time point were carried out for ~1 min, which provides ~600 individual ECG cycles for data analysis. To calculate HRV, we measured the time interval between all R waves (Fig. 3A). First, baseline HRV values were recorded and then mice were injected I.P. with atropine to determine the effects of parasympathetic blockade over 120 min. At baseline, *mdx* Sim 80 mice had a significantly greater HRV compared to *mdx* Con, as measured by the SD of R‐R intervals (Fig. 3B). Values for WT were on average also higher than *mdx* Con but did not reach statistical significance. After atropine injection, HRV rapidly decreased within 10 min for all groups (see Fig. 3B). HRV remained at this level for both *mdx* groups over 120 min but from 60 min after atropine injection, values increased for WT mice. Heart rate values were not significantly different between all groups at baseline nor at any time points after atropine administration (Fig. 3C).

### Simvastatin preserves cardiac function following long‐term treatment in *mdx* mice

**Figure 4:**
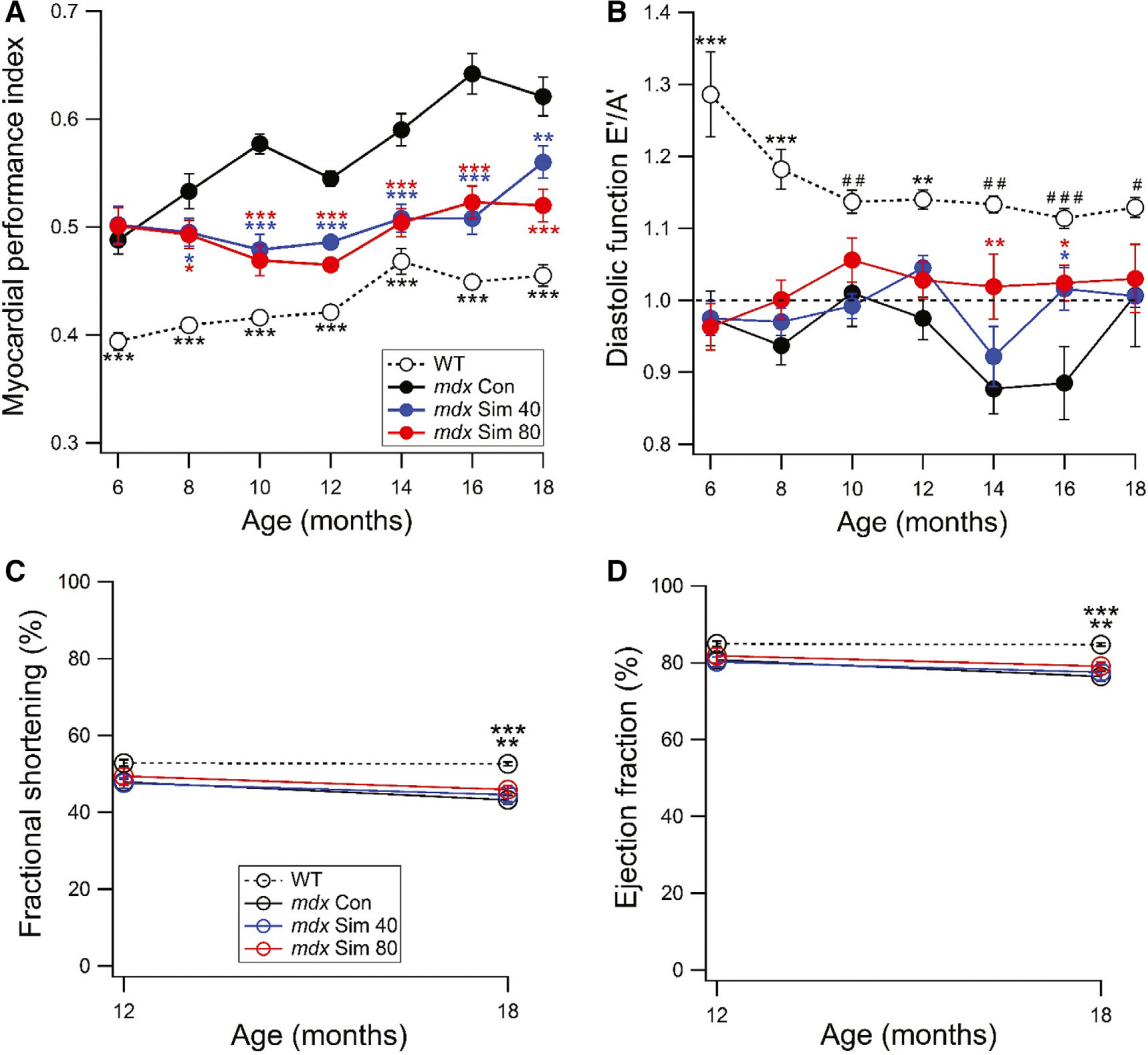
Long‐term simvastatin treatment provides sustained improvement of left ventricular diastolic function in *mdx* mice. In this study, *mdx* mice were treated with two doses of Simvastatin (40 or 80 mg/kg food) starting at 6 months of age for a total of 12 months treatment. WT mice were also used for this study. (A) Myocardial Performance Index (MPI), was measured by echocardiography every 2 months. The baseline measurement was taken when mice were 6 months of age (Time, 0 weeks). *P <* *0.05 versus *mdx* Con, **P <* *0.01 versus *mdx* Con, ***P <* *0.001 versus *mdx* Con, *P <* *0.05 versus *mdx* Con, ***P <* *0.001 versus *mdx* Con. ***P <* *0.001 versus all groups. (B) Diastolic function using the E’/A’ ratio. A ratio > 1.0 is considered a normal value (dotted line). *P <* *0.05 versus *mdx* Con, *P <* *0.05 versus *mdx* Con, **P <* *0.01 versus *mdx* Con, **P <* *0.01 versus all groups,***P <* *0.001 versus all groups, ^#^ *P *<* *0.05 versus *mdx* Con and *mdx* Sim 40, ^##^ *P *<* *0.01 versus *mdx* Con and *mdx* Sim 40, ^###^ *P *<* *0.001 versus *mdx* Con. At the start of the experiment, WT (*n* = 8), *mdx* Con (*n* = 13), *mdx* Sim 40 (*n* = 11), *mdx* Sim 80 (*n* = 12). Echocardiography measurements of (C) FS% and (D) EF% are shown at 12 and 18 months of age. **P <* *0.01 versus *mdx* Sim 80, ***P <* *0.001 versus *mdx* Con and *mdx* Sim 40.

In order to evaluate the effectiveness of simvastatin as a potential long‐term therapy for DMD, we next treated *mdx* mice for 12 months, from 6 to 18 months of age. For these studies, we compared two simvastatin doses, our established dose of 80 mg/kg food and half this dose (40 mg/kg food). Again, we focused on diastolic function, which we evaluated by echocardiography every two months. Interestingly, MPI values were already significantly higher at 6 months of age for *mdx* compared to WT mice (0.5 vs. 0.4, respectively) as shown in Figure 4A. This indicates that the onset of left ventricular diastolic dysfunction occurs at a relatively young age in *mdx* mice. Over time, MPI progressively increased for *mdx* Con mice, while simvastatin‐treated *mdx* mice maintained values over 12 months that were similar to the baseline at 6 months of age. WT mice showed a gradual increase in MPI over time but values always remained significantly lower than *mdx* Con and Sim mice (see Fig. 4A). We also assessed the E’/A’ ratio as an additional measure of diastolic function in these experiments. Here, values for *mdx* Con mice were slightly below 1.0 until 14 and 16 months of age, at which time there was a sharp reduction (Fig. 4B). At these time points, *mdx* Sim 80 mice showed a significant difference compared to *mdx* Con, while values for the *mdx* Sim 40 mice were also significantly higher than *mdx* Con at 16 months of age (see Fig. 4B). The increase in the E’/A’ back to 1.0 in 18‐month‐old *mdx* mice is likely due to a worsening of left ventricular diastolic function, which is associated with a compensatory reversal of the E’/A’ ratio (Panesar and Burch 2017) (see Discussion). Overall, this data indicates that simvastatin treatment provides long‐term benefits to cardiac function in *mdx* mice by preserving diastolic function. We also measured systolic function parameters, FS% (Fig. 4C) and EF% (Fig. 4D), at 12 and 18 months of age. There was no significant difference between any groups for both FS% and EF% at 12 months of age. At 18 months of age, there was a small but significant decrease in FS% and EF% for all *mdx* groups compared to WT.

### Simvastatin prevents cardiac fibrosis following long‐term treatment in *mdx* mice

**Figure 5:**
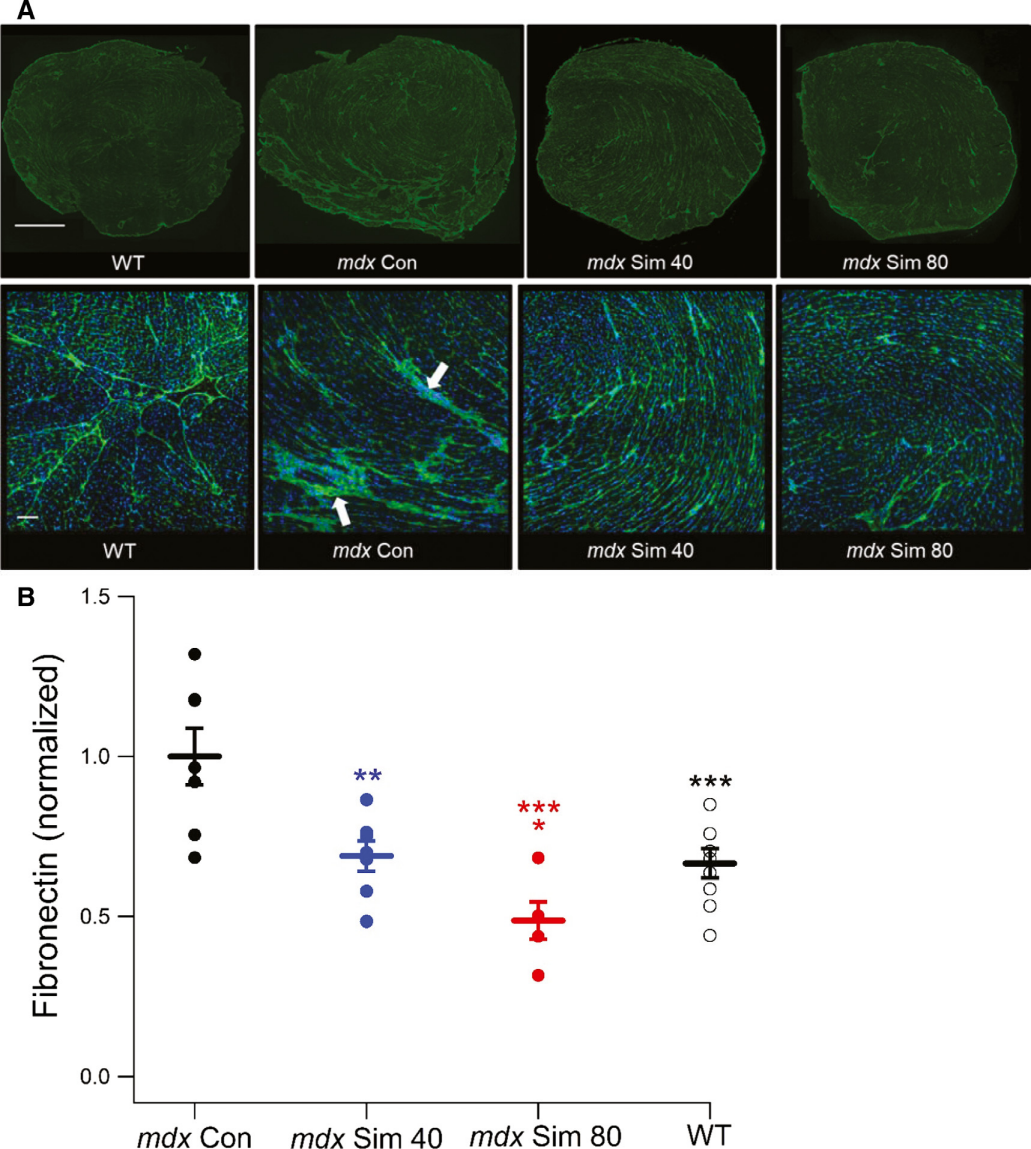
Long‐term simvastatin treatment prevents fibrosis in cardiac muscle of *mdx* mice. (A) Representative images of fibronectin immunostaining (green) from cardiac muscle cross‐sections of 18‐month‐old WT,* mdx* Con and *mdx* Sim‐treated mice following 12 months of simvastatin treatment. Nuclei are labeled with DAPI (blue). The upper panel shows whole heart cross‐sections, while the lower panel shows a selected area at higher magnification. Scale bars, upper panel = 1 mm, lower panel = 100 *μ*m. Note the patches of fibrosis in the *mdx* Con section (arrows). (B) Quantification of fibronectin immunofluorescence from cardiac muscle cross‐sections, showing individual and mean values. **P <* *0.01 versus *mdx* Con, *P <* *0.05 versus *mdx* Sim 40, ***P <* *0.001 versus *mdx* Con, ***P <* *0.001 versus *mdx* Con. WT (*n* = 10), *mdx* Con (*n* = 7), *mdx* Sim 40 (*n* = 7), *mdx* Sim 80 (*n* = 5).

The deposition of fibrotic connective tissue is an early pathogenic process in DMD cardiac muscle (Thomas et al. 2015) and therefore a key therapeutic target. Here, we evaluated the effect of long‐term simvastatin treatment on cardiac fibrosis in 18‐month‐old *mdx* mice by quantifying fibronectin immunofluorescence, a reliable method for evaluating fibrosis in *mdx* skeletal muscles (Percival et al. 2012; Whitehead et al. 2015). In cardiac muscle cross‐sections from *mdx* Con mice, numerous patches of fibronectin staining were observed, indicative of myocardial fibrosis (Fig. 5A). These patches of fibronectin staining were largely absent in sections from both *mdx* Sim and WT mice (Fig. 5A, arrows). Quantification revealed a significantly lower level of fibronectin for *mdx* Sim compared to *mdx* Con mice (Fig. 5B) with *mdx* Sim 80 displaying a greater reduction (50%) than *mdx* Sim 40 (31%). Importantly, fibronectin values for both simvastatin doses were not significantly different to WT mice (34% reduction compared to *mdx* Con) indicating that simvastatin prevented the development of pathological fibrosis in *mdx* cardiac muscle.

## Discussion

DMD is a debilitating, degenerative muscle disease, which currently lacks an effective long‐term treatment. As DMD patients are living longer due to improved ventilatory care, cardiac failure is now a major cause of death. Therefore, new therapies must address the need to effectively treat cardiac function in addition to improving the pathophysiology of skeletal muscle. Recently, we reported that simvastatin provided a dramatic improvement in skeletal muscle function of *mdx* mice due to the effective targeting of oxidative stress, inflammation and fibrosis (Whitehead et al. 2015). In the current work, our main aim was to determine if simvastatin could similarly provide improvements in the physiological function of dystrophic cardiac muscle. The key finding of our study was that treating *mdx* mice with simvastatin, at a low to moderate equivalent human dose (Whitehead et al. 2015), significantly improved overall left ventricular function in vivo, both in the short and long‐term. The main benefits provided by simvastatin included sustained improvement in diastolic function, increased HRV by enhanced parasympathetic activity, and the prevention of cardiac fibrosis.

Cardiac disease in DMD manifests as early diastolic dysfunction, which later progresses to dilated cardiomyopathy. Therefore, initially, our aim was to determine by echocardiography whether simvastatin could reverse early diastolic dysfunction in *mdx* mice. We used MPI as the main measure of left ventricular function since it is increased in 12‐month‐old *mdx* mice (Adamo et al. 2010) and young DMD patients before signs of overt cardiomyopathy (Shabanian et al. 2011). Furthermore, a recent study validated the utility of MPI for assessment of early, pre‐clinical cardiac disease in DMD patients (Spurney et al. 2015). Remarkably, we found that simvastatin mediated a dramatic reversal of MPI with values not significantly different to WT after 8 weeks of treatment. This was accompanied by a concomitant increase in the E’/A’ ratio over the same period, substantiating the improved diastolic function by simvastatin. While MPI is not widely used in the DMD field, it is an established, reliable method for assessing cardiac function in children with idiopathic cardiomyopathy (Azevedo et al. 2008) and its accuracy is not influenced by variables such as blood pressure or ventricular geometry (Goroshi and Chand 2016).

Perturbations in Ca^2+^ handling of dystrophic cardiomyocytes lead to impaired relaxation, which is a major cause of diastolic dysfunction (Cheng et al. 2012). Unphosphorylated PLB is a major inhibitor of SERCA2a activity in cardiac muscle, while p‐PLB‐S16 relieves the inhibition of SERCA and enhances activity of the Ca^2+^ pump (MacLennan and Kranias 2003). Here, we showed that p‐PLB‐S16 was significantly increased by simvastatin treatment compared to *mdx* Con, while total PLB and SERCA2a levels were not different. However, p‐PLB‐S16 was also increased in *mdx* Con compared to WT, consistent with a recent finding in younger *mdx* mice (Li et al. 2014). Interestingly, in support of our data, simvastatin was recently shown to increase p‐PLB‐S16 expression in rat ventricular cardiomyocytes (Pugh et al. 2014). While the underlying cellular mechanisms remain to be elucidated for this simvastatin‐mediated effect, it is likely that greatly increased p‐PLB‐S16 levels in cardiac muscle of *mdx* Sim mice lead to enhanced SERCA2a activity, thereby providing a possible mechanism for the improved diastolic function. In support of this idea, it was shown that transgenic expression of a pseudophosphorylated form of p‐PLB‐S16 greatly enhanced cardiac function in a dystrophic hamster model of dilated cardiomyopathy (Hoshijima et al. 2002).

Impaired regulation of the autonomic nervous system is another early pathway involved in cardiac dysfunction in DMD. This manifests as increased resting heart rate and decreased heart rate variability as well as increased probability of developing arrhythmias, which in the later stages of the disease may lead to sudden death (Smith et al. 2014). The autonomic dysfunction in DMD and *mdx* mice is characterized by both increased sympathetic and decreased parasympathetic activity (Smith et al. 2014). Interestingly, there is evidence that inhibition of sympathetic activity by beta blockers and ACE inhibitors does not improve the HRV in DMD (Thomas et al. 2015; Alvarez et al. 2017). There is, however, evidence that impaired parasympathetic activity mediates decreased HRV in DMD. Therefore, we decided to focus on the role of the parasympathetic nervous system in terms of its regulation of HRV in *mdx* mice. Importantly, we showed that simvastatin treatment for 2 months significantly increased baseline HRV in *mdx* mice. We then showed that atropine caused a rapid reduction in HRV for all groups of mice. This result indicates that parasympathetic activity has a major impact on HRV in mice and is most likely responsible for the greater HRV mediated by simvastatin. Another noteworthy finding was the recovery of HRV after 2 h in WT mice but not in simvastatin‐treated or untreated *mdx* mice. This finding suggests that the cellular pathway(s) by which simvastatin increased parasympathetic regulation of HRV in *mdx* mice is differentially regulated in WT mice. While this mechanism currently remains unknown, there is evidence that simvastatin improves autonomic function, including enhancement of parasympathetic activity, in patients with hypercholesterolemia (Brasileiro‐Santos et al. 2013). This finding is interesting since DMD patients (Srivastava et al. 2010) and *mdx* mice (Whitehead et al. 2015) have increased circulating cholesterol levels.

In order to be a viable treatment for DMD, long‐term efficacy is required since disease progression occurs over many years. Therefore, we treated *mdx* mice with simvastatin over 12 months, which is about half the entire lifespan of a mouse. Interestingly, we found that MPI was already higher in *mdx* mice at 6 months of age, indicating that diastolic dysfunction occurs at an even earlier age than previously known using this method (Adamo et al. 2010). Over time, MPI progressively increased in *mdx* Con mice but this was significantly reduced by both doses of simvastatin. We also measured the E’/A’ ratio in these studies and showed a significant increase for *mdx* Sim compared to *mdx* Con at 14 and 16 months of age. However, overall, our data from these long‐term studies indicate that the E'/A’ ratio is not as reliable as MPI, for accurate assessment of cardiac diastolic dysfunction progression in *mdx* mice. This is highlighted by the sharp increase in the E’/A’ value (0.9–1.0) for *mdx* Con mice from 16 to 18 months of age despite a worsening cardiac function as measured by MPI (see Fig. 4B). It is well known that the E’/A’ ratio depends on the stage of diastolic dysfunction (Mottram and Marwick 2005). During the early stages, values drop below 1.0, however as diastolic dysfunction progressively deteriorates, there is actually an increase in values due to a ‘pseudonormal’ or restrictive filling pattern (Mottram and Marwick 2005). This situation complicates the interpretation of E’/A’ measurements in older *mdx* mice as diastolic cardiac function worsens and therefore MPI is a more viable measure during this time.

Another key finding of the long‐term study was the prevention of fibrosis in *mdx* hearts following simvastatin treatment. Fibrosis is a major pathological mechanism leading to diastolic cardiac dysfunction in a wide range of diseases (Moreo et al. 2009), including young DMD patients (Thomas et al. 2015; Silva et al. 2017) and older *mdx* mice (Williams and Allen 2007). Remarkably, we showed that following long‐term treatment with simvastatin, fibronectin levels were the same as WT, indicating inhibition of fibrotic deposition in treated *mdx* mice. Previously we also showed reduced fibrosis in diaphragm muscles of simvastatin‐treated mice (Whitehead et al. 2015). We postulate that preventing fibrosis with simvastatin is likely a key mechanism underlying the improved diastolic function in *mdx* mice. Our data are consistent with a study in a rabbit model of human hypertrophic cardiomyopathy, where simvastatin caused regression of cardiac fibrosis, which was accompanied by improved cardiac function (Patel et al. 2001). In future studies we aim to elucidate the cellular mechanisms responsible for this important statin‐mediated benefit.

In summary, our results reveal that both short and long‐term treatment of *mdx* mice with simvastatin provides a dramatic improvement in cardiac muscle physiological function. Importantly, simvastatin provided functional benefits both before the onset of cardiac dysfunction and during the early stages of cardiomyopathy. Targeting both skeletal and cardiac muscle is a key requirement for the effective treatment of DMD. From a translational perspective, several statins, including simvastatin, are approved both in the US and UK for the treatment of familial hypercholesterolemia in pediatric patients 10 years of age or older. However, statins have been used off‐label in children as young as 4 years of age with no reports of any significant side‐effects (Humphries et al. 2018). Thus, our findings indicate that simvastatin has great potential to be a safe, inexpensive, and readily available therapy for improving both cardiac and skeletal muscle (Whitehead et al. 2015) function in DMD.

## Conflict of Interest

None declared.
